# Superior Trunk Block Catheter and 2% Chloroprocaine as a Phrenic Sparing Approach for Awake Arthroscopic Acromioclavicular Joint Surgery: A Case Report

**DOI:** 10.7759/cureus.55761

**Published:** 2024-03-07

**Authors:** Franklin Wou, Madan Narayanan

**Affiliations:** 1 Anesthesiology and Critical Care, Frimley Health National Health Service (NHS) Foundation Trust, Surrey, GBR

**Keywords:** superior trunk block, local anaesthetic, awake patient surgery, arthroscopic shoulder surgery, ultrasound guide regional anaesthesia

## Abstract

Regional anaesthesia for shoulder surgery remains challenging in patients with pre-existing respiratory comorbidities. Various alternative phrenic sparing techniques have been described in the literature, but to our knowledge, none have explored the benefits of using short-acting local anaesthetics in combination to achieve surgical anaesthesia for awake surgery. This case report describes the successful use of the superior trunk block catheter, a relative phrenic sparing shoulder nerve block, and 2% chloroprocaine, a short-acting local anaesthetic, to provide surgical anaesthesia for awake shoulder surgery in a patient with severe respiratory disease.

## Introduction

Regional anaesthesia for arthroscopic shoulder surgery presents a significant challenge for patients with pre-existing respiratory comorbidities. The gold standard technique of interscalene block (ISB) has a high incidence of inadvertent ipsilateral phrenic nerve blockade (up to 100%) [1] due to the close proximity to the site of injection and/or migration of the local anaesthetic (LA) to the C3-C5 nerve roots. In a patient with severe respiratory disease this effect could be poorly tolerated and the patient may have a high risk of respiratory complications postoperatively. The duration of the phrenic palsy is transient and dependent on the type and mass of local anaesthetic used [2].

Various “phrenic sparing” approaches have been described in the literature [3], but the evidence of these techniques for providing adequate surgical anaesthesia in an awake patient is limited due to the complex innervation of the shoulder involving the suprascapular, lateral pectoral, axillary and subscapular nerves. One of these approaches is the superior trunk block, performed distal to the ISB after the C5 and C6 nerve roots unite and form the superior trunk We would like to report a novel approach using a superior trunk (ST) perineural catheter and 2% chloroprocaine, short-acting LA, to provide anaesthesia for awake arthroscopic acromioclavicular joint (ACJ) excision.

## Case presentation

A 59-year-old woman underwent left-sided arthroscopic ACJ excision for a diagnosis of ACJ osteoarthritis with rotator cuff syndrome. The patient had multiple complex medical co-morbidities including severe COPD (secondary to alpha-1 antitrypsin deficiency), previous left upper lobectomy for lung adenocarcinoma, body mass index of 41, chronic pain secondary to spinal stenosis requiring chronic opioid and pregabalin use, and a poor functional baseline with dyspnoea on minimal exertion. The patient was deemed high risk for both general anaesthetic (GA) and our standard volume ISB (10-15 ml), and after the risk benefits of various anaesthetic options were discussed, the patient opted to have the surgery awake. The anaesthetic plan was to perform a “relatively phrenic sparing” ST block and conscious analgosedation intraoperatively with surgery performed in the sitting position.

The peripheral nerve block was performed in the anaesthetic room. A 20-gauge peripheral intravenous line was inserted, and monitoring was established via electrocardiography, SpO2 measurement, and non-invasive blood pressure. Midazolam 1 mg was administered intravenously as conscious sedation for block performance. The patient was positioned 45° head up with her head rotated to the right. The skin was sterilised with 0.5% chlorhexidine gluconate, and a high-frequency 13-6 mHz linear probe (Sonosite X-Porte, Amsterdam, Netherlands) was used. 

**Figure 1:**
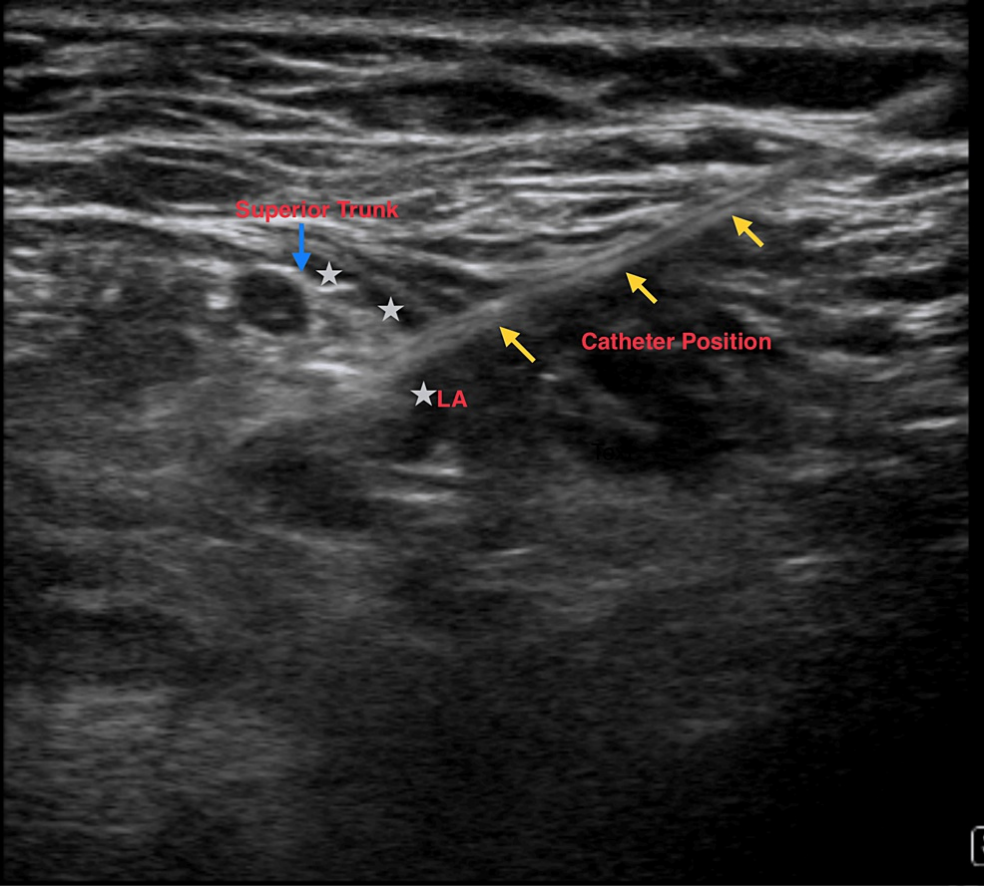
Sonography of the superior trunk nerve block using an in-plane technique The needle was inserted from a lateral to a medial approach. After the catheter was inserted, a local anaesthetic (LA) was injected and confirmed spread around the superior trunk.

The ST was located by first identifying the C5 and C6 nerve roots. These were traced inferiorly until they coalesced to form the ST. The skin was infiltrated with 1 ml of 1% lidocaine, and an ultrasound-guided lateral to medial in-plane ST block was performed using an 18-gauge 51 mm E-Cath Plus (Pajunk, Geisingen, Germany). Hydrodissection with normal saline confirmed the needle tip and subsequent catheter placement below the ST prior to the branching of the suprascapular nerve from the trunk (Figure 1).

Surgical anaesthesia was established with 2% chloroprocaine hydrochloride (Ampres®️Sintetica SA, Switzerland). An initial dose of 15 ml was given in titrated aliquots of 5 ml and adequate spread was noted below the ST. In addition, an in-plane ultrasound-guided superficial cervical plexus block was performed with 10 ml 0.25% bupivacaine under the posterior border of the sternocleidomastoid muscle at the level of C5.

Surgical anaesthesia was assessed from the confirmation of loss of cold sensation to ethyl chloride spray to C5 and C6 and motor weakness during shoulder abduction 20 minutes after the injection of local anaesthetic. The patient then mobilised and positioned herself in the beach chair position. 5 l of oxygen was provided as supplementary oxygen via a face mask and continuous capnography monitoring was started. Prior to the start of surgery, additional local infiltration was performed by the surgeon with 20 ml of 0.25% bupivacaine with adrenaline and an intravenous infusion of remifentanil (20 µg.ml-1) was commenced for analgosedation, titrated between 10-20 ml.hr-1. one hour after the original loading dose a further 5 ml of 2% chloroprocaine was given down the catheter.

The patient remained fully alert and haemodynamically stable throughout, only complaining of breakthrough pain towards the end of the operation, which was treated with 10-20 µg boluses of remifentanil. For postoperative analgesia, 10 ml of 0.25% bupivacaine was slowly given down the catheter towards the end of the operation in 2 ml aliquots. There were no complications of loss of verbal contact, apnoea, desaturations, or clinical evidence of respiratory distress from phrenic nerve palsy.

## Discussion

In this case report, it was demonstrated that the use a superior trunk catheter with a short-acting LA can provide adequate surgical anaesthesia for arthroscopic ACJ excision. The ST block was first described in 2014 as an alternative to the ISB in a patient with underlying pulmonary disease [4] and is considered a potential phrenic sparing nerve block for patients undergoing shoulder surgery [3]. Data on the risk of phrenic nerve blockade after an ST block or its near equivalent is very divergent. The risk of phrenic nerve palsy was less than 5% when using a two-point (above and below the trunk) single injection technique with 15 ml volume of 0.5% bupivacaine whilst providing equipotent surgical anaesthesia compared to the ISB [5], but a more recent study using a similar injection technique and volume of LA reported a rate of 54.3% for complete hemidiaphramatic paresis [6]. Whereas a cadaveric study aimed at blocking the suprascapular nerve anteriorly after the branch off from the superior trunk showed staining of the phrenic nerve in 20% of the dissections with a volume of 5 ml [7]. 

A low-dose or dilute ISB was also considered for this case but was decided against due to the risk of inadequate surgical anaesthesia and conversion to general anaesthetic intraoperatively and the relatively high risk of hemidiaphramatic paralysis of 33% even when using a volume of 5 ml [8].

The decision to use a catheter technique was chosen as it provided multiple advantages compared to a single shot. We hypothesised that the force of injection might play a factor in the tissue spread and phrenic nerve paresis. The catheter allowed the initial LA volume needed to establish surgical anaesthesia to be given over a longer period of time, potentially reducing the unintentional spread of the LA to the phrenic nerve and its roots. It also allowed further doses of LA to be given intraoperatively to extend surgical anaesthesia reducing the risk of conversion to general anaesthesia. Finally, the catheter allowed the ability to plan for postoperative analgesia either through a bolus or a continuous infusion of a longer acting LA. This was particularly beneficial for this patient given their history of chronic opioid use.

Chloroprocaine is an ester class LA indicated for use in peripheral nerve blocks. It has the advantage of providing fast onset and offset of anaesthesia due to rapid metabolism by pseudocholinesterases and low protein binding, with a duration of action of up to 60 minutes [9]. This unique pharmacology profile was the rationale behind its use in this case, as an adequate dose and volume can be given to provide reliable surgical anaesthesia for awake shoulder surgery, whilst the duration of any inadvertent phrenic palsy is limited by its short duration of action. This minimised the risk of prolonged phrenic nerve palsy and respiratory dysfunction postoperatively which would be poorly tolerated in patients with significant underlying respiratory disease. A previous study by Jafari et al. has demonstrated the benefit of chloroprocaine in reducing the time of onset of ISB when combined with bupivacaine [10], but no other studies have investigated its role as we described in limiting the effect of phrenic nerve palsy in shoulder blocks.

The patient was comfortable throughout the whole procedure and complained only of discomfort towards the end of the operation, likely due to our cautious approach to topping up the catheter intraoperatively to avoid phrenic nerve blockade and postoperative respiratory complication. Overall, the patient was satisfied with this anaesthetic approach and was discharged home the following day without any complications.

## Conclusions

This case report demonstrates that the combination of perineural superior trunk block catheter and 2% chloroprocaine can safely provide surgical anaesthesia for arthroscopic ACJ surgery in patients at high risk of respiratory complications. This technique can be considered as an alternative approach to phrenic-sparing nerve blocks when providing surgical anaesthesia for shoulder surgery, but further studies are needed looking at objective measurements of the incidence and duration of phrenic nerve palsy and reduction in diaphragm excursion when using this combination.
